# ZEB1 Regulates the Latent-Lytic Switch in Infection by Epstein-Barr Virus

**DOI:** 10.1371/journal.ppat.0030194

**Published:** 2007-12-14

**Authors:** Xianming Yu, Zhenxun Wang, Janet E Mertz

**Affiliations:** McArdle Laboratory for Cancer Research, University of Wisconsin School of Medicine and Public Health, Madison, Wisconsin, United States of America; Salk Institute for Biological Studies, United States of America

## Abstract

The immediate-early (IE) *BZLF1* gene of Epstein-Barr virus (EBV) regulates the switch between latent and lytic infection by EBV. We previously showed that the cellular transcription factor ZEB1 binds to a sequence element, ZV, located at nt −17 to −12 relative to the transcription initiation site of the *BZLF1* promoter, Zp, repressing transcription from Zp in a transient transfection assay. Here, we report the phenotype in the context of a whole EBV genome of a variant of EBV strain B95.8 containing a 2-bp substitution mutation in the ZV element of Zp that reduced, but did not eliminate, ZEB1 binding to Zp. Strikingly, epithelial 293 cells latently infected with the EBV ZV mutant spontaneously produced IE-, early-, and late-gene products and infectious virus, while wild-type (WT)-infected 293 cells did not and have never been reported to do so. Furthermore, treatment with the chemical inducers sodium butyrate and 12-*O*-tetradecanoyl-phorbol-13-acetate (TPA) led to an additional order-of-magnitude production of infectious virus in the ZV mutant–infected 293 cells, but still no virus in the WT-infected 293 cells. Similarly, ZV mutant–infected Burkitt's lymphoma BJAB cells accumulated at least 10-fold more EBV IE mRNAs than did WT-infected BJAB cells, with TPA or sodium butyrate treatment leading to an additional 5- to 10-fold accumulation of EBV IE mRNAs in the ZV mutant–infected cells. Thus, we conclude that ZEB1 binding to Zp plays a central role in regulating the latent-lytic switch in EBV-infected epithelial and B cells, suggesting ZEB1 as a target for lytic-induction therapies in EBV-associated malignancies.

## Author Summary

Ninety percent of people in the world become infected with Epstein-Barr virus (EBV). The virus can infect both epithelial and B cells, either making more virus and killing the cell or establishing a latent form of infection where it is stably maintained in the host. EBV infection is associated with the development of some types of cancer. We show here that a mere 2-bp substitution mutation in the silencer element, ZV, of the promoter of EBV's immediate-early *BZLF1* gene in the context of a whole EBV genome can lead to spontaneous reactivation of EBV out of latency into lytic replication, with production of infectious virus in some cells. The presence of the mutation also (i) made the virus more responsive to reactivation following treatment with chemical inducers, and (ii) disrupted binding of a cellular transcriptional repressor protein, ZEB1, to the BZLF1 promoter. Our work suggests a method to kill EBV-infected cancer cells by treating them with agents that lower the repressor activity of ZEB1. It also suggests one may be able to generate a vaccine against EBV infection using a constitutively lytic EBV strain made by knocking out the silencer elements of the BZLF1 promoter.

## Introduction

Epstein-Barr virus (EBV) is a human gamma herpesvirus estimated to infect 90% of the world's population. It is a causative agent of or associated with infectious mononucleosis and several B-cell and epithelial-cell cancers including Burkitt's lymphoma (BL), Hodgkin's disease, nasopharyngeal carcinoma, and some gastric carcinomas (reviewed in [1]). EBV can infect and immortalize primary B lymphocytes, leading to a latent form of infection in which its genome is maintained as an episome replicating via its viral origin of replication, *ori-P*, in synchrony with its host cell's DNA. EBV can also infect epithelial cells, leading to a latent or lytic form of infection depending upon the specific cell type and state of cellular differentiation. Only a few of the ∼100 genes in EBV are expressed during latency unless reactivation occurs, leading to lytic replication with production of infectious virus (reviewed in [2]).

Understanding regulation of the switch between latency and lytic replication is a central problem in herpes virology. EBV provides an excellent system to address this problem. Reactivation of EBV out of latency is usually initiated via activating expression of the immediate-early (IE) *BZLF1* gene [3] which encodes a sequence-specific DNA-binding protein, Zta (also called Z, Zebra, and EB1), a member of the bZIP family of leucine-zipper transactivators. The activities of Zta include directly participating in EBV replication via binding to the viral DNA origin of lytic replication, *ori-Lyt*, down-regulating the latency-associated promoters Cp and Wp, and serving as a transcriptional transactivator of its own promoter, other IE and early (E) viral promoters including the *BRLF1* promoter, Rp, and several cellular promoters. The *BRLF1* gene encodes a second viral transactivator, Rta (also called R). Acting together, Zta and Rta play multiple roles in lytic replication of EBV. While highly quiescent during latency, expression of the *BZLF1* promoter, Zp, can be activated in some cells by treatment with various inducers including phorbol esters such as 12-*O*-tetradecanoyl-phorbol-13-acetate (TPA), calcium ionophores such as ionomycin, sodium butyrate, transforming growth factor β (TGF-β) and anti-immunoglobulins (anti-Igs) (reviewed in [4]).

Transcription of the *BZLF1* gene can be initiated from either its proximal promoter, Zp, or the more distal promoter, Rp. However, most Zta protein is synthesized from mRNA initiated at Zp. The *cis*-acting elements of Zp sufficient for both basal and induced activity lie within the nucleotide (nt) −221 to +12 region relative to the promoter's transcription initiation site (reviewed in [4,5]) (Figure 1). Four AT-rich motifs, named ZIA through ZID, can bind the transcription factors Sp1, Sp3, and the myocyte enhancer factor 2D (MEF2D) [6,7]. ZII is a CRE-like motif that binds CREB, ATF family members, C/EBPs, and the AP-1 family of transcription factors [8–11]. ZIII contains multiple binding sites for the Zta protein itself [12]. Binding sites in Zp for Smads have also been identified [13].

**Figure 1:**
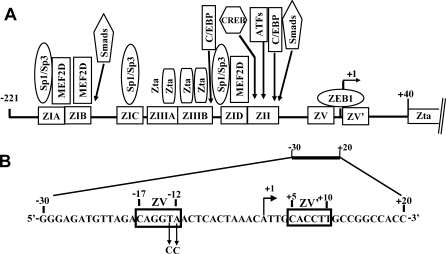
*BZLF1* Promoter (A) Schematic indicating the *cis*-acting regulatory elements present in the nt −221 through +40 region of Zp. Rectangles along Zp indicate approximate locations of known regulatory elements; their *trans*-acting factors are indicated above them. ZEB1 concurrently binds via its two zinc-finger domains to the two *cis*-acting elements ZV and ZV'. The transcription initiation site is indicated by a rightward arrow. (B) Nucleotide sequence of the nt −30 to +20 region of Zp. The ZV and ZV' elements are boxed. The 2-bp substitution mutation in the ZV element shown below the sequence was introduced here into the EBV genome.

Three elements involved in transcriptional silencing have also been identified within the nt −221 to +12 region of Zp: ZIIR [14], HIɛ [15], and ZV [16]. Other silencing elements, termed ZIV [17] and HIα-HIδ [15], lie within the nt −551 to −222 region of Zp. We have identified ZEB1 (also called δEF1, TCF8, AREB6, ZFHEP, NIL-2A, ZFHX1A, and BZP) as the main cellular factor in B-lymphocytes repressing transcription from Zp via binding the ZV element [18]. While repressors that recognize silencing elements upstream of nt −221 have been identified [19,20], ZEB1 and MEF2D are the only ones known to bind functionally within the nt −221 to +12 region of Zp.

Here, we report the effects of a 2-bp substitution mutation in the Zp ZV element in the context of a whole EBV genome. Quite strikingly, this ZV mutant (mt) of EBV spontaneously reactivated out of latently infected human epithelial 293 cells into lytic replication with production of infectious virus under conditions in which its WT parental virus never does. Burkitt's lymphoma BJAB cells latently infected with the ZV mutant also exhibited spontaneous synthesis of Zta mRNA under culture conditions in which WT-infected BJAB cells did not. Furthermore, treatment of the ZV mutant-infected BJAB cells with TPA led to high-level synthesis of Zta and Rta mRNAs while these IE viral genes remained silent in the WT-infected BJAB cells. Others have previously reported that ZEB1 is also a transcriptional repressor of the cellular E-cadherin promoter [21], with reduction in E-cadherin expression linked to tumor invasion, metastasis, and unfavorable prognosis [22]. ZEB1 represses by functioning as a molecular beacon for recruitment to promoters of CoREST-CtBP co-repressor complexes [23]. Taken together, we conclude that ZEB1 plays a central role in maintaining EBV infection in a latent form in both epithelial and B cells and, thus, is an excellent target for lytic-induction therapy for EBV-associated malignancies.

## Results

### Isolation of Cell Lines Latently Infected with a ZV Mutant of EBV

Substitution mutations in the Zp ZV element of either (i) A to C at nt −12 or (ii) A to C at nt −12 plus T to C at nt −13 relative to the Zp transcription initiation site were shown previously to partially relieve both (i) binding of ZEB1 and (ii) repression of Zp by ZEB1 in transient transfection assays [16,18] and mini-EBV replicons [24]. To examine the role of the Zp ZV element in establishing and maintaining EBV in a latent form of infection, we introduced this −12C, −13C 2-bp substitution mutation in the ZV element (ZVmt) into an intact EBV genome using a bacmid, p2089. Plasmid p2089 contains a wild-type copy of the complete genome of the B95.8 strain of EBV along with a mini F plasmid and expression cassettes for hygromycin phosphotransferase, green fluorescence protein (GFP), and chloramphenicol acetyl transferase [25]. The parental p2089-WT and two completely independent isolates of p2089-ZVmt were transfected in parallel into 293 cells, and the cells were grown thereafter in the presence of hygromycin. Numerous hygromycin-resistant, GFP-positive colonies were visible within 3–4 weeks after transfection with either p2089-WT or p2089-ZVmt. Independent clones of latently infected cells were grown out into cell lines. Virus stocks of WT and ZVmt EBV were generated by co-transfection of these cell lines with pCMV-BZLF1 and p2670, plasmids expressing the EBV-encoded proteins Zta and glycoprotein 110, respectively, to induce lytic replication and production of infectious virus [26]. The titers of these virus stocks were determined by a Raji cell assay [27]. Multiple independent cell lines containing p2089-WT or p2089-ZVmt which consistently yielded infectious virus with titers of 10^4^ to 10^5^ green Raji units (GRUs) per ml of medium following induction were chosen for further analysis.

To determine whether extraneous mutations might have arisen during either construction of the mutant plasmid or establishment and passage of the cell lines, p2089 DNA was extracted from each cell line. First, the nt −600 to +500 region of the *BZLF1* gene in each plasmid was PCR-amplified and resequenced to make sure no extraneous mutations were present in the promoter region of this gene; only the expected −12C, −13C substitution mutations were observed. Second, Southern blot analyses of *Bam*HI-digested plasmid DNA were performed with a variety of EBV DNA probes; no gross alterations in the EBV genomes were observed (e.g., Figure 2A; data not shown). The p2089 DNAs rescued from these cell lines were also digested with *Eco*RI, *Sal*I, and *Hin*dIII restriction endonuclease; again, no differences were observed in any of the restriction fragment digestion patterns compared to those of the original p2089 DNA (data not shown). Third, we also constructed a WT revertant, p2089-ZVmtRev, by introducing back into p2089-ZVmt DNA the original −12A and −13T sequence of Zp and isolated and extensively characterized three independent 293 cell lines latently infected with p2089-ZVmtRev. The phenotype of this revertant was found to be indistinguishable from that of the original p2089-WT in all assays performed on the latently infected 293 cells (see below). Thus, the EBV genomes present in these cell lines probably do not differ in any significant way from the B95.8 strain of EBV except for the 2-bp substitution mutation in the ZV element of Zp.

**Figure 2:**
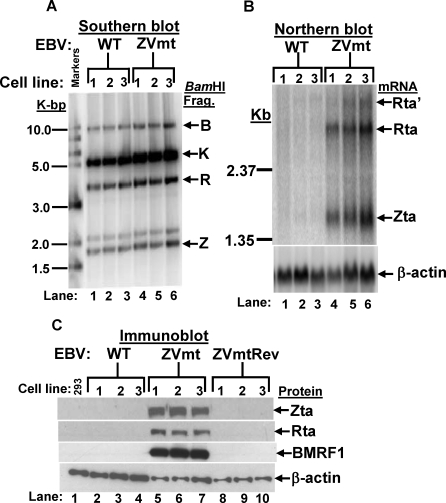
Presence of EBV DNA, mRNAs, and Proteins in the Latently Infected 293 Cell Lines Studied Here (A) Southern blot analyses of the *Bam*HI DNA fragments B, K, R, and Z of the p2089 DNAs isolated from the indicated WT- and ZV mutant-infected 293 cell lines. The locations of these *Bam*HI fragments are indicated on the right. (B) Mutation in the Zp ZV element leads to increased accumulation of Zta and Rta mRNA in latently infected 293 cell lines. Northern blot analyses were performed to detect the steady-state levels of Zta and Rta mRNAs present in the WT- and ZVmt-infected 293 cell lines. The bands corresponding to Rta, Zta, and β-actin mRNAs are indicated on the right. Rta' corresponds to the 4-kb, amino-terminally unspliced form of Rta mRNA. The size markers indicated on the left consisted of an RNA ladder (Invitrogen) run in the same gel. (C) Mutation of the Zp ZV element leads to increased synthesis of the EBV Zta, Rta, and BMRF1 proteins. Immunoblot analyses were performed with lysates of the indicated cell lines and antisera to Zta, Rta, BMRF1, and β-actin.

Three independent lines each of WT-, ZVmt-, and ZVmtRev-infected 293 cells were chosen for further detailed study. Two of the ZVmt-infected 293 cell lines were grown out from independent clones derived from cells transfected with one isolate of p2089-ZVmt DNA; the third was derived from cells transfected with p2089-ZVmt DNA obtained from a second, completely independent mutagenesis of p2089-WT DNA. Likewise, one of the three WT-infected 293 cell lines was the one originally described by Hammerschmidt and colleagues [25]. The other two were independent clones isolated from 293 cells transfected by us with p2089-WT DNA processed in parallel with the p2089-ZVmt DNAs constructed here.

### Spontaneous Reactivation of ZV Mutant EBV

293 cells latently infected with the B95.8 strain of EBV never spontaneously produce infectious virus because expression of the *BZLF1* and *BRLF1* genes is strongly repressed [28] (M. Altmann and W. Hammerschmidt, personal communication). Only one laboratory has reported observing WT-infected 293 cells spontaneously producing EBV [29]; even in this case, only 2% of over 250 clones examined produced any EBV, and the cells had been infected with the Akata strain of EBV, not the B95.8 strain. As expected, we, too, failed to detect expression of the EBV IE, E, or late (L) genes in any of our WT-infected 293 cell lines (Figures 2B, 2C, and 3). Strikingly, both Zta and Rta mRNAs were readily detected in all three ZVmt-infected 293 cell lines, present at levels at least 20-fold higher than in the WT-infected 293 cell lines (Figure 2B, lanes 4–6 vs. lanes 1–3). Immunoblot analyses confirmed expression of the IE genes, with each of the ZVmt-infected cell lines containing at least 30-fold more Zta, Rta, and BMRF1 protein than the WT- and ZVmtRev-infected cell lines (Figure 2C, lanes 5–7 vs. lanes 2–4 and lanes 8–10). BMRF1, an EBV E gene product, is only synthesized after Zta and Rta are present at sufficient levels to induce the later stages of the EBV lytic replication cycle. Immunofluorescence staining confirmed that Zta, Rta, and BMRF1 proteins were abundantly present in approximately 3% of the cells adhering to the dishes in each of the these three ZVmt-infected cell lines (Figure 3; data not shown). IFS also indicated the presence of gp350, a glycoprotein encoded by a late gene of EBV, in ∼1% of the cells (Figure 3; data not shown). Likely, once any ZVmt-infected cell expressed Zta at a level detectable in our IFS assay, it was destined to proceed on through the entire EBV lytic cycle of replication, eventually dying, detaching from the dish, and, thus, no longer being measured in our assay; we observed only one-third as many gp350-positive cells as Zta-positive cells because the former had fewer hours remaining before they detached from the dish. Our finding 3% Zta-positive cells is not a measurement of the percent of these mutant-infected cells able to reactivate; rather, it indicates these cells were spontaneously reactivating at a rate of ∼1% per day if one assumes the time from reactivation of the *BZLF1* promoter to cell death was ∼3 days. In contrast, we failed to detect any Zta-, Rta-, BMRF1-, or gp350-positive cells in any of the three WT-infected and three ZVmtRev-infected cell lines (Figure 3; data not shown). Thus, the 2-bp substitution mutation in the ZV element of Zp led to greatly increased expression of IE, E, and L genes of EBV in 293 cells. Therefore, we conclude that ZV mutant-infected 293 cells spontaneously reactivate into lytic replication under culture conditions in which 293 cells infected with the WT B95.8 strain of EBV have never been reported to do so.

**Figure 3:**
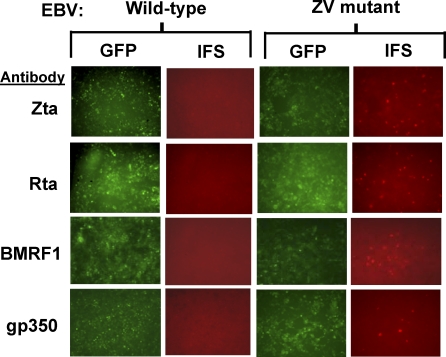
Indirect Immunofluorescence Staining (IFS) of 293-WT Cell Line 1 and 293-ZVmt Cell Line 1 for Presence of Zta, Rta, BMRF1, and gp350 Protein The primary antibodies used are indicated on the left of each row of images. Fields of cells were photographed with different filters to show the total EBV-positive ones (GFP) versus the subset of those containing the indicated EBV-encoded protein (IFS).

We also assayed the cell lines for production of infectious virus. Strikingly, all three ZVmt-infected cell lines spontaneously produced at least 10^2^ green Raji units (GRUs) per ml of medium (Figure 4A; Table 1). As expected, infectious virus was not detected in any of the three WT-infected and three ZVmtRev-infected cell lines unless the cells were transfected with an expression plasmid encoding Zta or Rta protein (e.g*.*, Figure 4A and Table 1; data not shown).

**Figure 4:**
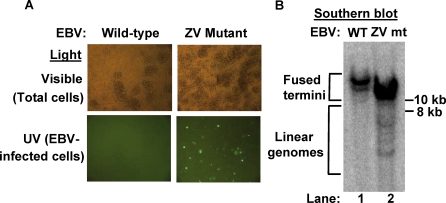
Production of Infectious Virus from the Latently Infected 293 Cell Lines Studied Here (A) Green Raji units (GRUs) assay showing production of infectious virus from 293-ZVmt cell line 1, but not 293-WT cell line 1. The EBV virus released into the medium was concentrated by centrifugation and used to infect Raji cells. Fields of Raji cells were examined with visible light (visible) for total cell number versus ultraviolet light (UV) for EBV-infected, GFP-positive cells. (B) EBV termini assay for presence of linear and circular episomal EBV genomes. Plasmid DNA was isolated from 293-p2089-WT cell line 1 and 293-ZVmt cell line 1, cleaved with *Bam*HI restriction endonuclease, separated by electrophoresis in a 0.8% agarose gel, transferred to a Hybond-N membrane (GE Healthcare), and probed with an EBV probe specific to the ends of the EBV genome [30]. The size markers indicated on the right consisted of a DNA ladder (New England Biolabs) run in the same gel. The positions of circular EBV genomes with fused termini and linear EBV genomes are indicated on the left.

**Table 1:**
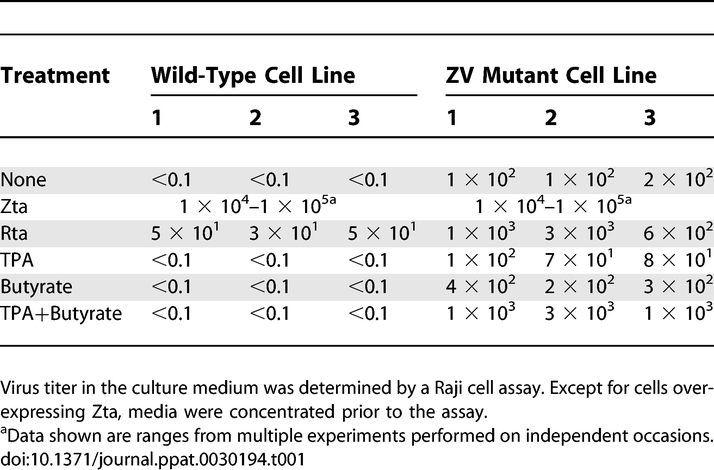
Spontaneous and Induced Reactivation of Wild-Type and ZV Mutant EBV in 293 Cells (GRU/ml)

To confirm that the results observed with the Raji cell assay were truly due to encapsidation of replicated viral DNA into infectious virion particles, we also examined the termini of the EBV genomes present in these cell lines. When EBV exists solely in a latent state as a circular episome, the ends of its linear viral genome are fused together; cleavage with *Bam*H1 restriction endonuclease generates large terminal EBV DNA fragments, somewhat heterogenous in size due to variability in the number of copies of a tandem repeated sequence present in this region of the EBV genome [30]. However, in lytically infected cells in which the EBV DNA has been linearized during packaging into virion particles, cleavage with *Bam*H1 generates smaller terminal EBV DNA fragments, also heterogeneous in size. As expected, the EBV DNA terminal *Bam*HI fragments isolated from the ZVmt-infected cell lines had sizes consistent with the presence of both circular and linear EBV genomes (e.g., Figure 4B; data not shown); no small EBV DNA terminal fragments were detected with the DNA isolated from the WT-infected cell lines (e.g., Figure 4B; data not shown). Therefore, we conclude that presence of the 2-bp mutation in the Zp ZV element led not only to derepression of Zp, but also to the entire subsequent series of events in the EBV lytic replication cycle necessary for production of infectious virus.

### Effects of Inducers on Reactivation Frequency

Transfection of cells with a Zta expression plasmid is the standard method for reactivating EBV-infected 293 cells into lytic replication for virus production [3] (Table 1). Exogenous expression of Rta can also reactivate EBV out of latency in some cell lines [31,32]. To determine whether ZVmt-infected 293 cells might be more susceptible to Rta induction than WT-infected ones because of partial derepression of Zp, we transfected them in parallel with a weak Rta expression plasmid, pEBV-RIE [31]. Both the WT- and ZVmt-infected 293 cell lines produced infectious virus following exogenous addition of Rta (Table 1). However, the virus titers obtained from the three ZVmt-infected cell lines were approximately 20-fold higher than they were from the three WT-infected cell lines. Immunoblot analysis indicated that these pEBV-RIE-transfected cells contained similar levels of Rta protein (data not shown). Thus, the significant difference in virus production was probably due to differences in endogenous expression of the *BZLF1* gene given that Zta and Rta function together in reactivation to lytic replication [33].

TPA and sodium butyrate are two well-known chemical inducers of latently infected B-lymphocytes. However, their presence, either individually or in combination, is not sufficient to reactivate the EBV B95.8 strain out of latency in 293 cells (M. Altmann and W. Hammerschmidt, personal communication; Table 1). Nevertheless, treatment of the ZVmt-infected 293 cell lines with these two chemicals in combination led to an additional ∼10-fold increase in production of infectious virus over the spontaneous rate observed with these cell lines (Table 1). Therefore, these two inducers synergized with the ZV mutation to yield a significantly higher frequency of reactivation of EBV than any of these three factors could accomplish by themselves or pairwise. Thus, TPA and sodium butyrate are probably primarily affecting positive and negative regulatory factors other than ZEB1 that also play roles in regulating Zp activity (e.g*.*, c-Jun, MEF2D). Reduction in ZEB1 binding to Zp, in combination with changes in these other regulators, is necessary to efficiently reactivate EBV to lytic replication in 293 cells. However, ZEB1 is a key, essential regulator since the ZV mutation, by itself, led to some reactivation, while treatment with TPA plus butyrate failed to lead to *any* virus production in WT-infected 293 cells.

### Effect of ZV Mutation on Zp Expression in BJAB Cells

BJAB is a human Burkitt's lymphoma cell line that can be efficiently infected by the B95.8 strain, with the EBV genome usually maintained as an episome [34,35]. To study whether our 2-bp substitution mutation in the ZV element also led to derepression of Zp in B-cells, we infected BJAB cells in parallel with WT and ZVmt virus. Hygromycin-resistant, GFP-positive clones were isolated and grown into cell lines. EBV DNA was isolated from each cell line and tested as described above by Southern blot analyses for (i) maintenance as an episome, (ii) average copy number per cell, and (iii) absence of gross sequence rearrangements (Figure 5A; data not shown). Two independent lines each of WT- and ZVmt-infected BJAB cells with similar episomal copy number were selected for further study.

**Figure 5:**
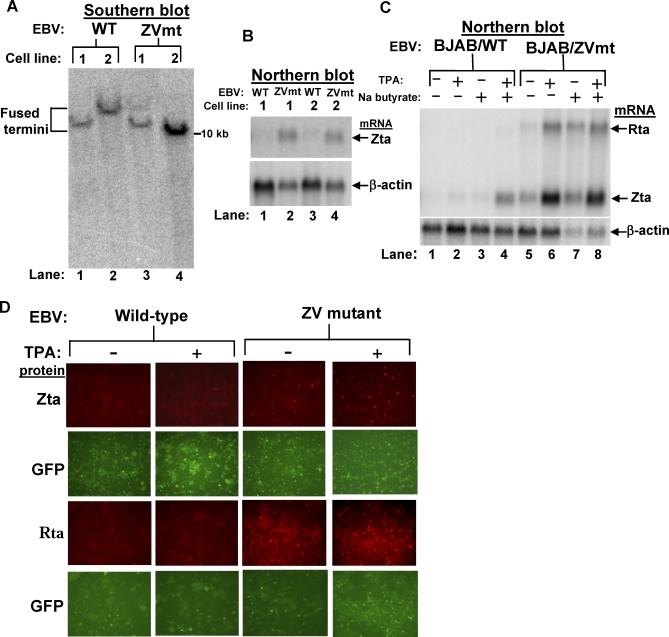
Effect of ZV Mutation in B cell Burkitt's Lymphoma BJAB Cells (A) Southern blot analysis of EBV DNA termini present in BJAB-WT and BJAB-ZVmt cell lines. (B) Northern blot analysis of Zta mRNA accumulated in BJAB cell lines latently infected with WT and ZVmt EBV. (C) Northern blot analysis of Zta and Rta mRNAs accumulated in BJAB-WT cell line 1 and BJAB-ZVmt cell line 1 following treatment with TPA or sodium butyrate as indicated for 48 h. The bands corresponding to Zta, Rta, and β-actin mRNAs are indicated on the right. (D) Immunofluorescence staining of BJAB-WT cell line 1 and BJAB-ZVmt cell line 1 for presence of Zta and Rta proteins. Cells were harvested and fixed 48 h after incubation in the absence or presence of TPA. Fields of cells were photographed with different filters to show the total EBV-positive ones (GFP) versus the subset of those containing the indicated EBV-encoded Zta and Rta proteins in the upper and lower panels, respectively.

As observed in 293 cells, the ZVmt-infected BJAB cell lines accumulated at least 10-fold more Zta mRNA than the WT-infected ones (Figure 5B, lanes 2 and 4 vs. lanes 1 and 3). Treatment of the mutant-infected cells with either TPA or sodium butyrate led to an additional 5- to 10-fold accumulation of Zta mRNA; treatment in combination led to a synergetic, 15- to 20-fold increase in accumulation (Figure 5C, lanes 5–8). On the other hand, treatment of the WT-infected BJAB cells with either TPA or sodium butyrate did not significantly affect accumulation of Zta mRNA; rather, significant activation of the WT promoter was only observed when these cells were treated concurrently with both inducers (Figure 5C, lane 4 vs. lanes 1–3). Immunofluorescence staining showed that Zta-positive and Rta-positive cells were readily detectable in the ZVmt-infected BJAB cell lines with or without TPA treatment, but not in the WT-infected BJAB cell lines (Figure 5D; Table 2).

**Table 2:**
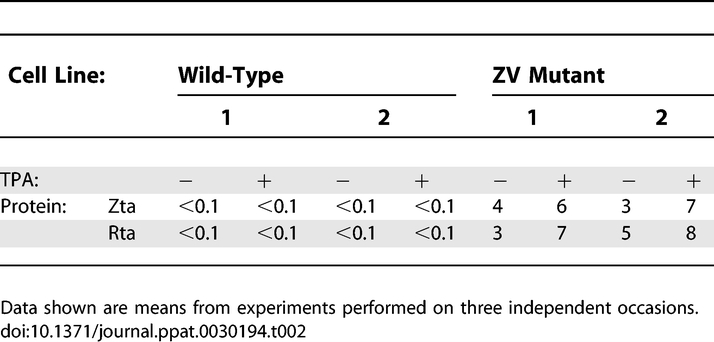
Percent Latently Infected BJAB Cells Synthesizing Zta and Rta Protein Spontaneously and After TPA Induction

Synthesis of Zta protein in the ZVmt-infected BJAB cells led to derepression of Rp, with significant synthesis of both Rta mRNA (Figure 5C, lane 1 vs. lane 5) and protein (Figure 5D) as well. Nevertheless, we failed to detect linear EBV DNA termini indicative of packaging of replicated viral DNA into virion particles in the ZVmt-infected BJAB cell lines (Figure 5A). However, given that neither over-expression of Zta nor treatment with chemical inducers leads to lytic replication in WT-infected BJAB cells [35], failure of the ZVmt-infected BJAB cells to undergo a complete lytic cycle of infection was expected. Regardless, we conclude that the 2-bp substitution mutation in the ZV element led to significant derepression of Zp in B-lymphocytic BJAB cells, with the ZVmt-infected BJAB cells being significantly more susceptible to additional induction of Zp by TPA and sodium butyrate than the WT-infected BJAB cells. These findings are very similar to the ones obtained with the WT- versus ZVmt-infected 293 cell lines (Figures 2 and 3; Table 1).

### ZEB1 Binds the ZV Element of Zp In Vivo

We performed quantitative chromatin immunoprecipitation (ChIP) assays to test directly whether ZEB1 is associated with Zp via the ZV element in 293 cells latently infected with EBV. Chromatin was isolated from formaldehyde-fixed 293, WT-infected 293, ZVmt-infected 293, and 293 cells infected with the wild-type revertant of the ZV mutant, ZVmtRev. The chromatin was sheared by sonication to fragments with an average length of ∼500 bp, and subjected to immunoprecipitation with a ZEB1-specific antibody. DNA extracted from the precipitated chromatin was used as template for quantitative PCR amplification with a pair of primers specific for Zp. As positive and negative controls, the PCR amplification was also performed with pairs of primers specific for (i) Rp, which also contains a ZEB1-binding site [18], (ii) the interleukin 2 promoter (IL-2p), a cellular promoter with a known ZEB1-binding site [36], and (iii) an EBV sequence located 4.8-kbp upstream of the Zp transcription initiation site. The ZV mutant exhibited a 2- to 3-fold reduction in ZEB1 binding to Zp compared with the parental WT and the revertant, i.e., the 2-bp mutation reduced but did not abolish ZEB1 binding (Figure 6A, lane 3 vs. lanes 2 and 4; see Figure 6B for quantitation). This finding was expected given our recent identification of a second consensus ZEB1-binding element in Zp, named ZV', located at nt +5 through +10 relative to the Zp transcription initiation site (Figure 1A; Yu, Wang, and Mertz, unpublished data). Also as expected, the ZV mutation had no effect on binding of ZEB1 to either Rp or IL-2p (Figure 6A, lane 3 vs. lanes 2 and 4; Figure 6B), and the assay was specific for ZEB1 antibody (Figure 6D) and DNA containing a ZEB1-binding site (Figure 6E). Thus, we conclude that the ZV element of Zp is a functional ZEB1-binding site. When ZEB1 is present in cells, it binds concurrently with very high affinity to both the ZV and ZV' elements of Zp via its two zinc-finger domains [37], thereby maximally silencing expression of the *BZLF1* gene to ensure latency is maintained. Mutation of the ZV element weakens binding of ZEB1 to Zp, enabling occasional transcription initiation from Zp and, thus, synthesis of Zta mRNA and protein, leading to reactivation and the subsequent cascade of events that result in lytic replication with synthesis of infectious virions.

**Figure 6:**
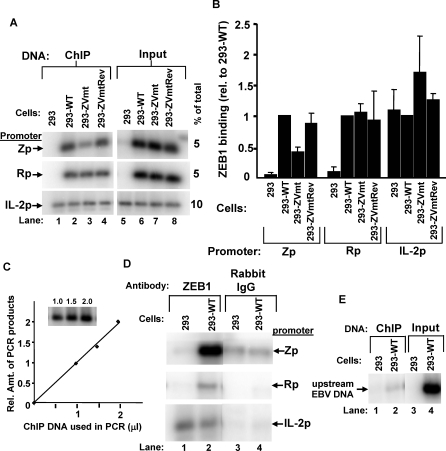
Quantitative ChIP Assay Showing Reduced Binding of ZEB1 to Zp in ZVmt-Infected 293 Cells (A) Autoradiogram of radiolabeled PCR products resolved by electrophoresis in a 2.5% agarose gel. Lanes 1–4, DNA templates were obtained from chromatin isolated from 293, 293-WT, 293-ZVmt, and 293-ZVmtRev cells immunoprecipitated with a ZEB1-specific antibody; and lanes 5–8, DNA templates were obtained from input DNA isolated from these cells prior to immunoprecipitation. The percent of the DNA sample used in each PCR amplification reaction is indicated on the right. (B) Summary of quantitative analysis of results of ChIP assays performed as shown in panel (A) on three separate occasions starting with cells harvested on different days. Data were normalized to the amount of PCR product obtained from 293-WT ChIP DNA and shown as means plus standard errors of the means. (C) Amplification of 1–2 μl of ChIP DNA by 25 cycles of PCR yields products in the linear range of the assay. The insert shows the autoradiogram of PCR products obtained starting with 1.0, 1.5, and 2.0 μl of ChIP DNA and the primers for Zp. (D) Autoradiogram of PCR products of DNA templates obtained by immunoprecipitation of chromatin from 293 and 293-WT cells with antibody to ZEB1 (lanes 1 and 2) versus pre-immune rabbit IgG (lanes 3 and 4). (E) Autoradiogram of PCR products from DNA templates obtained from chromatin immunoprecipitated with ZEB1 antibody (lanes 1 and 2) or input DNA isolated prior to immunoprecipitation (lanes 3 and 4) using a pair of primers corresponding to a region of EBV located 4.8-kbps upstream of the Zp transcription initiation site that lacks a ZEB1-binding site. The 190-bp PCR product is indicated by an arrow.

## Discussion

We examined here the effects of mutating the ZV element of Zp on maintaining latency and reactivating EBV in epithelial and BL cell lines. This is the first report of a systematic analysis of the role of a *cis*-acting transcriptional regulatory element of EBV within the context of a whole genome. We show that a 2-bp substitution mutation in the ZV element can have dramatic effects on the EBV life cycle. While this particular ZV mutant established latency in epithelial 293 cells at a frequency similar to WT EBV, it was defective in maintaining this latency. All three of the ZV mutant-infected cell lines we examined in detail spontaneously synthesized Zta mRNA and protein at levels sufficient to reactivate the virus into its lytic cycle, with synthesis of Rta, early proteins including BMRF1, late proteins including gp350, linear viral genomes, and infectious virus (Figures 2–4; Table 1). Neither we nor anyone else to the best of our knowledge has ever observed spontaneous reactivation of the WT B95.8 strain of EBV in laboratory-infected 293 cells. We also did not observe any spontaneous reactivation in 293 cells infected with the WT revertant of the ZV mutant (Figure 2C; data not shown). Strikingly, the ZV mutant-infected cell lines were also more susceptible to induction by either (i) over-expression of Rta, or (ii) treatment with TPA and sodium butyrate (Tables 1 and 2; Figure 5C). Quite likely, the phenotype of the ZEB1-binding site mutant would have been even more dramatic if the ZV element mutation had been combined with a ZV' element mutation to further reduce or completely eliminate ZEB1 binding to Zp. Furthermore, preliminary data indicate that the ZV mutant virus is at least an order-of-magnitude defective relative to WT EBV in establishing colonies of proliferating lymphocytic cells following infection of primary human B cells (Yu and Mertz, unpublished data). Thus, we conclude that the ZV element is a key component of Zp, playing a central role in regulating the switch in the EBV life cycle between latency and lytic replication.

We have recently published in collaboration with the Kenney laboratory that exogenous addition of ZEB1 represses Zp activity from both a Zp reporter plasmid and whole EBV genomes in gastric carcinoma ZEB1-negative AGS cells that are normally highly lytic for EBV [38]. Taken together with previously published data showing that numerous different mutations in the ZV element exhibit a similar effect on Zp activity in a transient transfection reporter assay [16,18], this finding provides strong evidence that the phenotype of the ZV mutant studied here is, indeed, due to it altering ZEB1 binding rather than fortuitously creating a binding site for another transcription factor.

Our finding that the ZV mutant can establish a latent form of infection in 293 cells rather than being a constitutive lytic mutant suggests that, although the ZEB1-binding ZV element is a physiologically important silencer of Zp, other negative regulatory elements (e.g*.*, ZEB1-binding ZV' element, MEF2D-binding ZI elements, ZIIR element [14]) also contribute to the very tight repression of Zp observed in 293 cells. In addition, for efficient reactivation of EBV from latency into lytic replication, positive transcriptional regulatory factors (e.g*.*, ZII-binding proteins such as ATFs, CREBs, and AP-1) are required as well. Thus, while ZEB1 binding to Zp plays a central role in regulating EBV's switch between latency and lytic replication, it functions in coordination with other cellular regulators of Zp to determine whether the *BZLF1* gene is expressed in a specific cell type under specific growth conditions. Consistent with this conclusion, we have recently published in collaboration with the Kenney laboratory data that indicate the following: (i) ZEB1 levels range from very high to moderate to absent among a variety of epithelial and B-cell types that are physiologically relevant to EBV; (ii) The correlation between ZEB1 abundance and whether infection by EBV is latent or lytic is fairly good, but far from perfect; and (iii) The abundance of activated c-Jun also contributes to how a cell responds to EBV infection [38]. In addition, loss of Zp repression via ZEB1 binding to the ZV/ZV' elements might also occur via changes in ZEB1 interactions with cellular co-regulators leading to ZEB1, itself, switching from a repressor binding CtBPs to an activator binding Smads, p300, and other co-activators [39].

Sodium butyrate inhibits the activity of histone deacetylase complexes (HDACs), leading to acetylation of histone proteins and, subsequently, promoters becoming more accessible to transcriptional activators and the cellular transcription machinery [40]. TPA is a multi-functional inducer. It activates the cellular protein kinase C pathway in B cells [41], inducing phosphorylation of Zta protein [42] which can then activate Rp [43] leading to synthesis of Rta protein and subsequent activation of Zp [44]. Nevertheless, treatment of WT-infected 293 cell lines with TPA plus sodium butyrate fails to induce reactivation of EBV (e.g., Table 1). In contrast, identical treatment of the ZVmt-infected 293 cell lines led to a 10-fold increase in virus production above that observed spontaneously (Table 1). Thus, high-affinity binding of ZEB1 to the ZV/ZV' elements of Zp directly over the transcription initiation site is sufficient to maintain repression of the *BZLF1* gene expression in 293 cells even when inducers make Zp much more accessible to its positive regulators. With the 2-bp mutation in the ZV element studied here partially alleviating binding of ZEB1 to Zp (Figure 6), the addition of these inducers increased the probability that transcription from Zp would reach the threshold level at which Zta protein was produced in sufficient quantity to irreversibly activate transcription from Zp and Rp, leading to full-blown reactivation with lytic replication.

Binding of ZEB1 to Zp was also found to be important in B cells. Zp expression was tightly repressed and not inducible by either TPA or sodium butyrate in the WT-infected B-lymphocytic BJAB cells. Only treatment of the WT-infected BJAB cells with both inducers in combination led to significant accumulation of Zta mRNA (Figure 5C). On the other hand, Zp was spontaneously expressed in the ZV mutant-infected BJAB cells. Moreover, either TPA or sodium butyrate was sufficient to further activate Zp expression, with significant accumulation of Rta as well as Zta mRNA, presumably due to Zta protein activating Rp expression. Unfortunately, since BJAB cells do not support EBV late gene expression and viral DNA replication, up-regulation of Zp and Rp in the mutant-infected BJAB cells did not lead to production of infectious virus. Nevertheless, our recent preliminary finding that the ZV mutant virus is at least an order-of-magnitude defective relative to the parental WT EBV in establishing colonies of proliferating lymphocytic cells following infection of primary human B cells (Yu and Mertz, unpublished data) provides strong evidence that ZEB1 binding to the ZV element of Zp is, indeed, important for establishing a stable latent infection in B cells as well as epithelial cells.

We found that the ZV mutant exhibited only a 2- to 3-fold reduction in ZEB1 binding to Zp (Figure 6), yet a 20-fold or more increase in accumulation of Zta mRNA and protein (Figures 2 and 5). These two sets of data are quite compatible given the fact that a small initial increase in expression of the *BZLF1* gene in the context of cells infected with whole EBV genomes can lead to a large change in expression because Zta protein activates expression of the *BRLF1* gene, with the Rta protein then up-regulating *BZLF1* gene expression to high levels. These findings also provide an explanation for why we chronically observe a small percentage of the ZVmt-infected 293 cells to have spontaneously reactivated into full-blown EBV lytic replication while most of the cells in the population still score as Zta-negative in our IFS assay (Figure 3), i.e*.*, once a cell manages to synthesize Zta mRNA and protein above a critical threshold level, it irreversibly switches into the lytic cycle. On the other hand, transcription of Zp remains tightly repressed in the WT-infected 293 cells, with infectious virus never produced without exogenous addition of Zta or Rta (e.g., Table 1).

Amon et al. [45] noted little difference by immunoblot analysis between wild-type and the 2-bp ZV mutant of Zp studied here in *BZLF1* gene expression before versus after anti-Ig induction in Akata cells. This seeming discrepancy with our results could be due to epigenetic (e.g., methylation status of Zp) or structural (e.g*.*, Zp's distance from *OriP*) differences between the p2089 bacmid used by us and the mini-EBV plasmid used in their study. Alternatively, their use of more sensitive assays (e.g., qRT-PCR, different method for extraction and detection of Zta protein) likely would have revealed that the ZV mutant actually did over-express Zp, leading to higher accumulation of Zta mRNA and protein at earlier times after anti-Ig induction, consistent with the large difference in Zp expression they had reported earlier between wild-type and the ZV mutant using a luciferase reporter assay [24].

In summary, we show that a mere 2-bp substitution mutation in the ZEB1-binding site in Zp in the context of a whole EBV genome can lead to expression of EBV IE, E, and L genes and production of infectious virus under conditions in which the B95.8 strain of WT EBV has never been observed to reactivate out of latency. Given this finding, we speculate that loss of ZEB1 binding to Zp as a repressor by (i) mutation of the ZV' as well as the ZV element of Zp, (ii) knock down of synthesis of ZEB1 in the host cell, or (iii) switching ZEB1 from a repressor to activator of transcription (e.g., via TGF-β signaling of Smads [39]) could lead to efficient reactivation into lytic EBV replication in latently infected cells, especially if combined with loss as well of other repressors of *BZLF1* gene expression (e.g*.*, MEF2D, the ZIIR element-binding protein) and activation of transcriptional activators (e.g*.*, c-Jun). Thus, ZEB1 is a novel candidate target for lytic-induction therapies for some EBV-associated malignancies [46]. In addition, an EBV strain containing mutations in the ZV/ZV' elements in combination with a mutation in another negative regulatory element of Zp (e.g*.*, ZIIR) may provide the basis for development of a constitutively lytic strain of EBV that could serve as a vaccine for immunization against EBV infection.

## Materials and Methods

### Cells and plasmids.

293, a human embryonic kidney cell line, was obtained from W. Hammerschmidt. Raji and BJAB are EBV-positive and -negative human BL cell lines, respectively. They were maintained at 37 °C in RPMI1640 medium supplemented with 10% fetal bovine serum (FBS).

Plasmid pCMV-BZLF1 [47], encoding Zta protein, and plasmid p154.13, containing nt ∼101,000 through ∼113,000 of EBV strain B95.8, were obtained from B. Sugden. Plasmid p2089 [25], containing the complete genome of EBV B95.8 strain, and plasmid p2670 [26], encoding EBV glycoprotein gp110, were obtained from W. Hammerschmidt. Plasmid pGS284, containing an ampicillin cassette and the levansucrase gene for positive and negative selection, respectively, and E. coli strains GS500 (RecA+) and S17λpir [48] were provided by S. Speck. Plasmid pEBV-RIE [31], encoding Rta protein, was obtained from S. Kenney. Plasmids containing the *Xho*I and *Eco*RI subfragments of EBV for termini assays were provided by N. Raab-Traub [30].

### Mutagenesis of p2089.

The 2-bp substitution mutation, ZVmt, was introduced into the Zp ZV element in p2089 by allelic exchange in E. coli as described by Smith and Enquist [49] and Moorman et al*.* [48]. Briefly, the mutation was generated by a two-step, PCR-based site-directed mutagenesis of plasmid p154.13. A 1,100-bp EBV DNA fragment containing the mutated ZV element near its center was cloned into pGS284 and transformed into S17λpir by electroporation (Bio-Rad). Plasmid p2089 was transformed by electroporation into GS500. The resulting S17λpir and GS500 cells were mated. Integration of pGS284 into p2089 and its subsequent excision via homologous recombination were sequentially selected using the markers present in these two plasmids. PCR screening indicated 5% of the p2089 revertants had lost the WT Zp and retained the ZVmt one. The desired mutation in p2089 and nowhere else throughout the entire *BZLF1* promoter region was confirmed by DNA sequence analysis. The p2089-ZVmt clones were also digested with a variety of restriction enzymes including *Eco*RI, *Bam*H1, *Hin*dIII, and *SaI*I. Only ones with digestion patterns identical to the parental p2089 were retained. A wild-type revertant of p2089-ZVmt, p2089-ZVmtRev, was constructed by mutagenesis of p2089-ZVmt and thoroughly characterized likewise.

### Isolation of WT- and ZVmt-infected 293 and BJAB cell lines.

The p2089-WT and p2089-ZVmt DNAs were purified by equilibrium centrifugation in CsCl-ethidium bromide gradients, introduced into 293 cells with Lipofectamine 2,000 (Invitrogen), and selected by incubation in the presence of 100 μg/ml hygromycin as described by Neuhierl et al*.* [26]. GFP-positive colonies were picked 4- to 6-weeks later and grown into cell lines. To make virus stocks, these EBV-infected cells were co-transfected with pCMV-BZLF1 (5 μg/100-mm dish) and p2670 (5 μg/100-mm dish) using Lipofectamine 2000. The culture medium was harvested 72 h later, passed through a 0.8-μm filter, and stored at 4 °C.

BJAB cells were infected with WT and ZV mutant virus stocks at a multiplicity of infection of 0.1 GRUs per cell as described by Marchini et al*.* [35], plated at 100 cells per well in 96-well plates, and grown in the presence of 300 μg/ml hygromycin until GFP-positive colonies emerged. The EBV genomes in each of the numerous 293 and BJAB cell lines were extensively characterized by DNA sequence analysis of the entire *BZLF1* promoter region and restriction fragment patterns as described above.

### Northern and Southern blot analyses.

Whole-cell polyadenylated RNA was isolated with oligo(dT) cellulose [50]. In Figure 5C, mRNA was harvested after incubation of the cells with TPA and sodium butyrate for 48 h. Northern blot analysis was performed as previously described [51]. Radiolabeled probes were prepared using a random primer labeling system (Amersham). A 311-bp fragment from nt +8 to +318 relative to the Zp transcription initiation site, generated by PCR, was used as template for making the probe for detection of both Zta and Rta mRNAs. A 177-bp human β-actin fragment [52], a gift from S. Guang, was used as template for making a β-actin probe. Southern blot analysis was performed as previously described [53]. The p2089 DNA was digested with *Bam*HI prior to electrophoresis in a 0.8% agarose gel. Plasmid p154.13 was used as template for making a probe that detects EBV's *Bam*HI fragments Z, R, K, and B. EBV termini assays were performed as previously described [30].

### Immunoblot analyses and IFS.

To detect Zta, Rta, and BMRF1 proteins by immunoblotting, cells in 100-mm dishes were lysed in SUMO lysis buffer [54]. EBV proteins were separated by SDS-12% PAGE and detected by incubation with monoclonal anti-Zta (Argene, 1:200 dilution), anti-Rta (Argene, 1:100 dilution), anti-BMRF1 (Capricorn, 1:100 dilution), or anti-β-actin (Sigma, 1:5000 dilution) antibodies, followed by incubation with goat anti-mouse IgG horseradish-conjugated secondary antibody (Pierce, 1:5,000 dilution). The bound secondary antibody was visualized using a chemiluminescence kit (Roche). For IFS, the cells were fixed with methanol:acetone (1:1) for 10 min at room temperature, pre-incubated with phosphate-buffered saline (PBS) containing 20% FBS, incubated with monoclonal anti-Zta (1:40 dilution), anti-Rta (1:40 dilution), anti-BMRF1 (1:40 dilution), or anti-gp350 (Chemicon, 1:100 dilution) antibody, washed with PBS, incubated with a Texas Red-conjugated, anti-mouse IgG secondary antibody (Jackson Laboratories, 1:100), washed with PBS, embedded in mounting medium (Vector Laboratories), and examined with a fluorescence microscope (Zeiss).

### Assays for infectious EBV.

To assay for spontaneous reactivation, latently infected 293 cells were plated in 100-mm dishes and incubated for 3 days until ∼80% confluent. The medium was harvested, passing through a 0.8-mm filter, and adjusted to 8 ml total volume. For chemical induction, the cells were incubated for 48 h with TPA (20 ng/ml), sodium butyrate (3 mM), or both before the medium was processed as above. For induction with Rta, the cells were transfected with pEBV-RIE (5 μg per 100-mm dish) with Lipofectamine 2000. The relative virus titers were determined by a Raji cell assay, with the number of GFP-positive cells being counted in a hemocytometer by ultraviolet microscopy [27]. This assay underestimates the concentration of infectious virus by a factor of at least 10 [27]. When virus titer was low, the virus particles were concentrated by centrifugation in an 80Ti Beckman rotor for 2 h at 17,500 rpm prior to infection of the Raji cells.

### ChIP assay.

Quantitative ChIP analysis was performed as described by Aparicio et al. [55]. Briefly, 293 cells latently infected with WT, ZV mutant, or ZVmtRev along with uninfected 293 cells as a negative control were fixed with formaldehyde and used to prepare chromatin. After sonication to shear the chromatin to average size of ∼500 bp, antibody to ZEB1 (Santa Cruz Biotechnology) and protein A/G plus-agarose beads (Santa Cruz Biotechnology) were added to immunoprecipitate the ZEB1-containing chromatin. The beads were collected by centrifugation and washed extensively. The cross-linked protein-DNA complexes were eluted and heated at 67 °C to reverse the crosslinking. The resulting DNA was subject to 25 cycles of PCR using the following primers: Zp, forward 5′-TGATGTCATGGTTTGGGA-3′, reverse 5′-CTGCATGCCATGCATA-3′; Rp, forward 5′-GGGTGGTGATGTAGCTATAC-3′, reverse 5′-CCTAGGGATTTCATAAAGGCC-3′; IL-2p, forward 5′-CTACATCCATTCAGTCAGTC-3′, reverse 5′-AACTCTTGAACAAGAGATGC-3′; and negative control EBV sequence located 4.8-kbp upstream of the Zp transcription initiation site, forward 5′-AGAAGGGAGACACATCTGGA-3′, reverse 5′-AACTTGGACGTTTTTGGGGT-3′. Controls included the following: (i) The PCR condition used (94 °C 30 sec, 55 °C 30 sec, 72 °C 1 min for 25 cycles) was one experimentally determined to yield products falling within the linear range of the assay (Figure 6C); (ii) Normal preimmune rabbit IgG immunoprecipitated at most one-fifth of the level of Zp DNA (Figure 6D); and (iii) ZEB1 antibody failed to immunoprecipitate EBV sequences located 4.8-kbps upstream of the Zp transcription initiation site (Figure 6E). The PCR products were resolved by electrophoresis in a 2.5% NuSieve 3:1 agarose gel (Cambrex) and the resulting DNA bands obtained from three independent sets of experiments were quantified using a PhosphorImager (Figure 6B).

## Supporting Information

### Accession Numbers

Accession numbers at the NCBI (http://www.ncbi.nlm.nih.gov/) database for human ZEB1 (TCF8) and the EBV genome strain B95.8 are U12170 and V01555, respectively.

## Abbreviations

anti-Ig: anti-immunoglobulin
EBV: Epstein-Barr virus
GFP: green fluorescence protein
GRU: green Raji unit
IE: immediate-early
IFS: immunofluorescence staining
MEF2D: myocyte enhancer factor 2D
mt: mutation
qRT-PCR: quantitative reverse transcription-PCR
Rev: revertant
TGF-β: transforming growth factor β
TPA: 12-*O*-tetradecanoyl-phorbol-13-acetate
WT: wild-type
ZEB1: zinc finger E-box binding protein 1
